# Empowering adolescent girls through video-assisted self-defense teaching program in rural Gujarat, India

**DOI:** 10.6026/973206300200728

**Published:** 2024-07-31

**Authors:** Mahalakshmi B., Sivasubramanian N., Limbachiya Jaiminkumar Bipinbhai, Thakor Ankitaben Babuji, Krishnamoorthy M.R., Mothliya Prashviben Devajibhai, Jadav Kajalben Pravinbhai

**Affiliations:** 1 Nootan College of Nursing, Sankalchand Patel University, Visnagar, Gujarat - 384315, India; 2 Velammal college of Nursing, Madurai, Tamilnadu- 625009, India

**Keywords:** Adolescent girls, self-defense education, gender-based violence

## Abstract

Adolescent girls face myriad challenges impacting their mental health and well-being, necessitating empowerment through self-defense education. In contexts of prevalent gender-based violence, such education becomes imperative, particularly in countries like India. However, the influence of demographic factors on knowledge levels regarding self-defense techniques among adolescent girls remains uncertain. This study employed a one-group pre-test - post-test design to evaluate the impact of a video-assisted self-defense teaching program on adolescent girls in rural Gujarat, India. A sample of 100 girls from Mahesana district schools participated, with data collected via structured questionnaires administered pre and post-intervention. The intervention significantly enhanced participants' knowledge levels, with a remarkable increase in mean post-test scores compared to pre-test scores. Specifically, prior to the intervention, 45% of participants exhibited low knowledge levels, which improved to 19.5% post-intervention. Notably, 80.5% demonstrated excellent knowledge post-intervention. The study underscores the efficacy of a video-assisted self-defense teaching program in augmenting knowledge levels among adolescent girls in rural Gujarat. Despite demographic diversity, the intervention yielded consistent improvements, emphasizing its universal applicability.

## Background:

Teenage girls encounter a myriad of challenges that can significantly impact their mental health and overall well-being. From bullying to insecurity, these issues can lead to feelings of depression and vulnerability, often resulting in substance abuse and a loss of self-esteem. The detrimental effects of depression on mental health are well-documented, with studies even suggesting potential implications for physical health, such as growth inhibitory effects on melanoma cells. In the face of such adversities, self-defense emerges as a crucial countermeasure aimed at safeguarding the health and well-being of adolescent girls. [1] Self-defense techniques, systematically taught in defense classes, offer practical strategies for addressing threats of violence and harassment that teenage girls commonly encounter. These techniques not only provide physical protection but also empower girls to assert their boundaries and advocate for their safety. [2] Moreover, the benefits of self-defense extend beyond mere physical skills, with studies indicating its potential to reduce stress and enhance mental resilience, particularly in the context of human epidermal keratinocytes. [3] In today's world, where incidents of sexual assault and harassment are alarmingly prevalent, self-defense education has become increasingly imperative, especially for teenage girls. The pervasiveness of gender-based violence underscores the urgent need to equip girls with the knowledge and skills to protect themselves in various settings, including schools, streets, and even within their own homes. Recognizing the significance of self-defense in addressing gender violence, efforts are being made to integrate self-defense education into formal schooling systems, highlighting its status as an essential component of holistic education. [4] Eve teasing, a pervasive form of sexual harassment, poses a significant threat to the safety and well-being of adolescent girls, both in urban and rural areas. Studies indicate that girls are frequently subjected to eve teasing, further emphasizing the importance of self-defense training as a means of prevention and empowerment. Additionally, self-defense serves as a proactive measure to prepare girls for unexpected situations, fostering increased mental and physical health. [5] In a society where women, often perceived as the weaker sex, are considered easy targets for perpetrators of violence, self-defense assumes paramount importance. With statistics revealing the high risk of women falling victim to violent crimes, empowering girls with self-defense skills becomes not only practical but also ethical. In a country like India, where cases of gender violence are escalating, self-defense education for women is no longer a luxury but a necessity. [6] It is essential to acknowledge that teaching self-defense may not be suitable for every individual, and the decision to pursue self-defense training should be made with careful consideration. Nevertheless, in a world where the safety and well-being of women are constantly under threat, self-defense emerges as a vital tool for empowerment and protection. By addressing the root causes of insecurity and providing support for mental health, we can create a safer and more equitable environment for teenage girls to thrive and fulfill their potential.

## Methodology:

## Research design:

The research design employed for this study was a one-group pre-test - post-test design [7]. This design involves measuring the participants' knowledge levels before and after the intervention to assess the effectiveness of the intervention in enhancing their understanding of self-defense techniques.

## Sample and sample size:

The sample consisted of 100 adolescent girls selected from schools in Mahesana district, Gujarat, India. These participants were chosen based on their availability and consent to participate in the study.

## Sampling technique:

Convenience sampling was utilized to select the participants for the study [8]. This sampling technique was chosen for its practicality and ease of access to the target population within the selected schools in Mahesana district.

## Data collection:

Data were collected using a structured questionnaire administered to the participants before and after the intervention. The questionnaire included items assessing demographic information such as age, class of study, mother's education, type of family, and previous knowledge of self-defense techniques. Additionally, pre-test and post-test sections were included to evaluate the participants' knowledge levels regarding self-defense techniques before and after the intervention.

## Statistical analysis:

Descriptive statistics, such as frequencies and percentages, were used to summarize demographic characteristics and knowledge levels of the participants. Inferential statistics, including paired t- were employed to examine the differences in knowledge scores before and after the intervention. The Chi-square test was used to determine the significance of associations between categorical variables, such as age, education level, and knowledge regarding self-defense techniques. Analysis was conducted using statistical software such as SPSS to determine the effectiveness of the intervention in enhancing participants' knowledge of self-defense techniques.

## Results:

The demographic characteristics of the participants are summarized in Table 1. The majority of the participants belonged to the age group of 14.1 to 16 years; comprising 50% of the total sample, while 22.5% were aged 14 and 27.5% were aged 16.1 to 18 years. Regarding religion, 94% of the participants identified as Hindu, 6% as Muslim, and none as other religions. In terms of education, the largest proportion of mothers had upper primary education (42.5%), followed by primary education (33.75%), while 12.5% were illiterate and 11.25% were graduates. Additionally, 55% of the participants belonged to joint families, and 70% resided in rural areas. Regarding pre-test and post-test knowledge scores of the participants, prior to the intervention 45% of the participants exhibited low knowledge levels, while 55% had average knowledge. Following the video-assisted teaching program, there was a notable improvement in knowledge levels, with 19.5% of participants demonstrating average knowledge and 80.5% exhibiting excellent knowledge. The mean pre-test knowledge score was 7.75, while the mean post-test score significantly increased to 14.42. This resulted in a mean difference of -6.68, indicating a substantial improvement in knowledge levels after the intervention. The standard deviation for the pre-test was 1.87, and for the post-test, it was 1.59. The calculated t-value of 27.45 indicates a statistically significant difference between the pre-test and post-test knowledge scores.

The association between demographic variables and knowledge levels regarding self-defense techniques among adolescent girls was examined using Chi-square tests. Results revealed non-significant associations between age [χ^2^ = 0.808, p > 0.05], religion [χ^2^ = 0.485, p > 0.05], standard [χ^2^ = 2.228, p > 0.05], mother's education [χ^2^ = 2.925, p > 0.05], type of family [χ^2^ = 0.661, p > 0.05], number of siblings [χ^2^ = 0.779, p > 0.05], area of residence [χ^2^ = 0.779, p > 0.05], and previous knowledge [χ^2^ = 1.117, p > 0.05] with knowledge levels. These results suggest that factors such as age group, religious affiliation, educational background, family structure, and residential area did not significantly influence the girls' understanding of self-defense techniques. Overall, the findings indicate that demographic characteristics may not be strong determinants of knowledge levels in this context.

## Discussion:

The findings of our study reveal a significant improvement in knowledge levels among participants regarding self-defense techniques after the implementation of a video-assisted teaching program. Prior to the intervention, a considerable proportion of participants exhibited low knowledge levels, but post-intervention, the majority demonstrated excellent knowledge. This substantial improvement is reflected in the mean pre-test and post-test knowledge scores, with a notable increase observed after the intervention. The statistical analysis further confirmed the efficacy of the intervention, showing a significant difference between pre-test and post-test scores. In comparison to similar studies, our findings align with the results reported by Nanthili *et al.* (2016), where a structured educational intervention led to a significant improvement in knowledge levels among participants. [9] Similarly, Susila *et al.* (2021) observed a considerable increase in knowledge scores following an educational program targeting self-defense techniques. [10] These studies underscore the importance of targeted interventions, such as video-assisted teaching programs, in enhancing knowledge and skills related to self-defense among adolescent girls.

Regarding the association between demographic variables and knowledge levels, our study found non-significant associations between various demographic factors and knowledge levels that aligns with study conducted by Agarkhed *et al.* [11] This contrasts with the findings of other studies, such as Chavan *et al.* (2020) [12] and Jordan *et al.* [13] where certain demographic variables were found to be significantly associated with knowledge levels. However, our study suggests that factors such as age group, religious affiliation, educational background, family structure, and residential area may not significantly influence knowledge levels in this context. These findings highlight the importance of context-specific interventions tailored to the target population's needs. While demographic factors may play a role in determining knowledge levels in some studies, our results suggest that other factors, such as the effectiveness of the educational intervention itself, may have a more significant impact on knowledge acquisition. Further research is needed to explore additional factors that may influence knowledge levels regarding self-defense techniques among adolescent girls and to inform the development of more effective educational programs in this area.

## Figures and Tables

**Figure 1:**
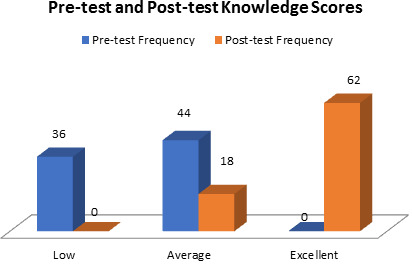
Frequency distribution of sample as per their knowledge category in pre-test and post test

**Table 1:** Demographic Characteristics of Participants

**Demographic Variable**	**Category**	**Frequency**	**Percentage**
Age	14	18	22.5
	14.1 to 16	40	50
	16.1 to 18	22	27.5
Religion	Hindu	75	94
	Muslim	5	6
	Other	0	0
Standard	8	11	13.75
	9	13	16.25
	10	56	70
Mother's Education	Illiterate	10	12.5
	Primary Education	27	33.75
	Upper Primary	34	42.5
	Graduate	9	11.25
Type of Family	Nuclear	36	45
	Joint	44	55
Number of Siblings	No Any	12	15
	One	28	35
	Two	24	30
	More than Two	16	20
Area of Residence	Urban	24	30
	Rural	56	70
Previous Knowledge	Yes	58	72.5
	No	22	27.5
